# Single-cell spatial atlas of smoking-induced changes in human gingival tissues

**DOI:** 10.1038/s41368-025-00385-5

**Published:** 2025-08-01

**Authors:** Yong Zhang, Zongshan Shen, Jiayu Yang, Junxian Ren, Chi Zhang, Lingping Tan, Li Gao, Chuanjiang Zhao

**Affiliations:** Hospital of Stomatology, Guangdong Provincial Key Laboratory of Stomatology, Guanghua School of Stomatology, Sun Yat-sen University, Guangzhou, China

**Keywords:** RNA sequencing, RNA sequencing, Periodontitis

## Abstract

Smoking is a well-established risk factor for periodontitis, yet the precise mechanisms by which smoking contributes to periodontal disease remain poorly understood. Recent advances in spatial transcriptomics have enabled a deeper exploration of the periodontal tissue microenvironment at single-cell resolution, offering new opportunities to investigate these mechanisms. In this study, we utilized Visium HD single-cell spatial transcriptomics to profile gingival tissues from 12 individuals, including those with periodontitis, those with smoking-associated periodontitis, and healthy controls. Our analysis revealed that smoking disrupts the epithelial barrier integrity, induces fibroblast alterations, and dysregulates fibroblast–epithelial cell communication, thereby exacerbating periodontitis. The spatial analysis showed that endothelial cells and macrophages are in close proximity and interact, which further promotes the progression of smoking-induced periodontal disease. Importantly, we found that targeting the endothelial CXCL12 signalling pathway in smoking-associated periodontitis reduced the proinflammatory macrophage phenotype, alleviated epithelial inflammation, and reduced alveolar bone resorption. These findings provide novel insights into the pathogenesis of smoking-associated periodontitis and highlight the potential of targeting the endothelial–macrophage interaction as a therapeutic strategy. Furthermore, this study establishes an essential information resource for investigating the effects of smoking on periodontitis, providing a foundation for future research and therapeutic development for this prevalent and debilitating disease.

## Introduction

Periodontitis is a highly prevalent chronic inflammatory disease affecting the supporting tissues of teeth, ultimately leading to tooth loss if left untreated; it is characterized by complex interactions between bacterial infection and the host immune response, resulting in the destruction of periodontal tissues.^1,2^ Smoking is a major risk factor for periodontitis and significantly impacts disease progression and treatment outcomes.^3^ Compared with nonsmokers, smokers exhibit more severe periodontal destruction, increased alveolar bone loss, and a poorer response to periodontal therapy.^4^ Considering that 22.3% of the global population smokes and approximately 1.1 billion people are smokers,^5^ thus, exploring the mechanisms of smoking-induced periodontal damage and developing precise therapeutic strategies are highly important.

The detrimental effects of smoking on periodontal health are attributed to various mechanisms, including an altered immune response, altered endothelial cell function, and the disruption of epithelial‒fibroblast communication.^6–8^ Studies have shown that smoking can significantly alter the immune response in periodontal tissues, affecting various cell types, including epithelial cells, fibroblasts, and immune cells.^3,9,10^ Smoking not only alters gene expression but is also believed to trigger abnormal immune responses.^11^ Despite extensive research on smoking-associated periodontitis, the precise mechanisms by which smoking affects the periodontal tissue microenvironment remain incompletely understood. Previous studies have focused primarily on individual cell types or specific molecular markers, lacking the comprehensive spatial context that modern technologies can provide.

Recent advances in spatial transcriptomics technology have revolutionized our understanding of tissue architecture and cellular interactions in various diseases.^12^ This technology enables the simultaneous analysis of gene expression patterns while preserving spatial information, providing unprecedented insights into the complex tissue microenvironment.^13^ Despite these advancements, current spatial transcriptomic technologies are often limited by their inability to achieve single-cell resolution or by their reliance on fresh-frozen tissue samples.^14^ As a result, gaining a comprehensive understanding of tissue organization, particularly in the gingiva, using biobanked samples has remained a challenge.^15^ Additionally, given the complex effects of smoking on cell–cell interactions within the periodontal tissue microenvironment,^3,16^ spatial transcriptomics with single-cell resolution offers a unique advantage. Unlike traditional single-cell sequencing,^17^ it not only captures gene expression profiles but also provides crucial spatial information on smoking-induced changes in human gingival tissues.

Therefore, the aim of this study was to utilize high-resolution spatial transcriptomics to comprehensively characterize the impact of smoking on the periodontal tissue microenvironment. By employing the Visium HD platform, we sought to map the spatial distribution of different cell types within healthy and diseased periodontal tissues and identify smoking-induced changes in gene expression patterns across various cell populations. Moreover, understanding the complex cellular interactions that contribute to disease progression in smoking-associated periodontitis is important. Furthermore, the results of this study confirmed that targeting aberrant CXCL12 signalling in endothelial cells alleviates smoking-associated periodontal inflammation and bone destruction. This comprehensive analysis provides valuable insights into the pathogenesis of smoking-associated periodontitis and may identify novel therapeutic targets for treatment.

## Results

### Construction of a single-cell resolution spatial transcriptomics atlas of human gingivae

To construct a single-cell resolution spatial transcriptomics atlas, gingivae were collected from eight patients with periodontitis, four with periodontitis (P), four with smoking-associated periodontitis (SP), and healthy gingivae (HG) were collected from four healthy volunteers. Formalin-fixed paraffin-embedded (FFPE) tissue blocks were generated from these samples (*P* = 4, SP = 4, HG = 4). Sections of the FFPE tissue blocks were processed for Visium HD-based analysis to investigate the effects of smoking on the periodontal tissue microenvironment.

**Fig. 1:**
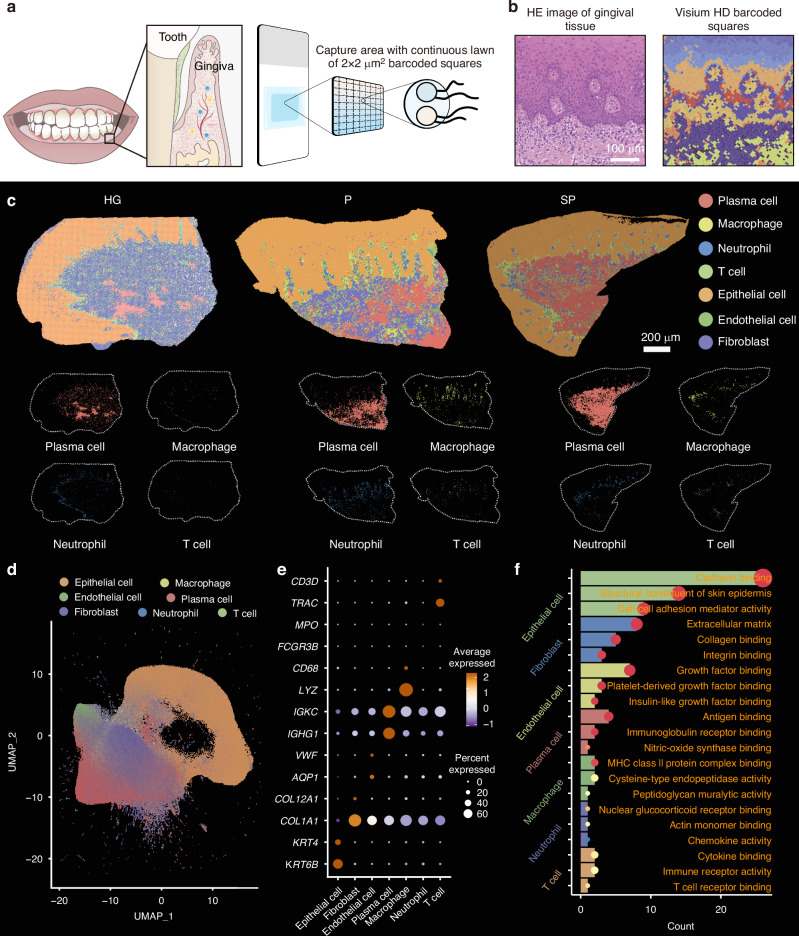
Construction of a single-cell resolution spatial transcriptomic atlas of human gingivae. **a** Schematic diagram of the single-cell resolution spatial transcriptomic analysis. **b** Image of H&E-stained gingival tissue and cell clustering of Visium HD data. **c** Identification of 7 cell types in the gingival tissues of the healthy gingivae (HG), periodontitis (P) and smoking-associated periodontitis (SP) groups. **d** UMAP plots showing single-cell clustering of the gingival tissue, displaying 7 major cell types. **e** Dot plot showing the expression of specific marker genes in 7 major cell types. The colour of the dot represents the expression level of the marker genes in each cell. High expression levels are shown in brown, and low expression levels are shown in blue. **f** GO analysis depicting the functions of the marker genes for each cell type

The Visium HD platform allows for high-resolution spatial gene expression analysis using single-cell resolution probes targeting the entire transcriptome. Visium HD slides are structured with a reference frame of 8 × 8 mm, containing a capture area of 6.5 × 6.5 mm. Each capture region comprises approximately 11 million 2 × 2 µm squares arranged in a continuous array of unique barcode oligonucleotides. These 2 µm squares are directly adjacent to each other, forming a continuous and gap-free capture region (Fig. 1a). The high-resolution data generated by Visium HD were used to perform unsupervised clustering, identifying distinct cell subpopulations and mapping those populations onto the morphological features of the gingival tissue (Fig. 1b).

The cells were categorized into four main groups—epithelial cells, fibroblasts, endothelial cells, and immune cells—through integration and clustering techniques. Subsequent single-cell transcriptomic analysis combined with spatial transcriptomics allowed further classification of immune cells into T cells, plasma cells, macrophages, and neutrophils, which were then projected onto the gingival tissue morphology (Fig. 1c). Cluster analysis was conducted, and the cell populations were visualized on a uniform manifold approximation and projection (UMAP) plot (Fig. 1d and Supplementary Fig. 1) using data from 250 000 cells after quality control.

Marker gene expression levels were assessed for each cell group and depicted in dot plots using R software to identify specific marker genes for each cell type: epithelial cells (*KRT4* and *KRT6B*), fibroblasts (*COL1A1* and *COL12A1*), endothelial cells (*VWF* and *AQP1*), plasma cells (*IGHG1* and *IGKC*), macrophages (*CD68* and *LYZ*), neutrophils (*FCGR3B* and *MPO*), and T cells (*TRAC* and *CD3D*) (Fig. 1e). A pathway enrichment analysis was conducted on the marker genes within each cell group. Marker genes of epithelial cells were associated with structural components of the skin epidermis and cell–cell adhesion mediator activity; fibroblast marker genes were linked to extracellular matrix and collagen binding; endothelial cell marker genes were related to growth factor binding and platelet-derived growth factor binding; plasma cell marker genes were related to antigen binding and immunoglobulin receptor binding; macrophage marker genes were associated with MHC class II protein complex binding and cysteine-type endopeptidase activity; neutrophil marker genes were related to chemokine activity and actin monomer binding; and T cell marker genes were linked to immune receptor activity and T cell receptor binding(Fig. 1f). These results were consistent with the typical functional pathways of these cell subpopulations. The single-cell resolution spatial transcriptomics atlas of human gingivae obtained here provides the basis for further analyses of cellular changes under smoking conditions.

### Smoking-induced damage in gingival epithelial cells

**Fig. 2:**
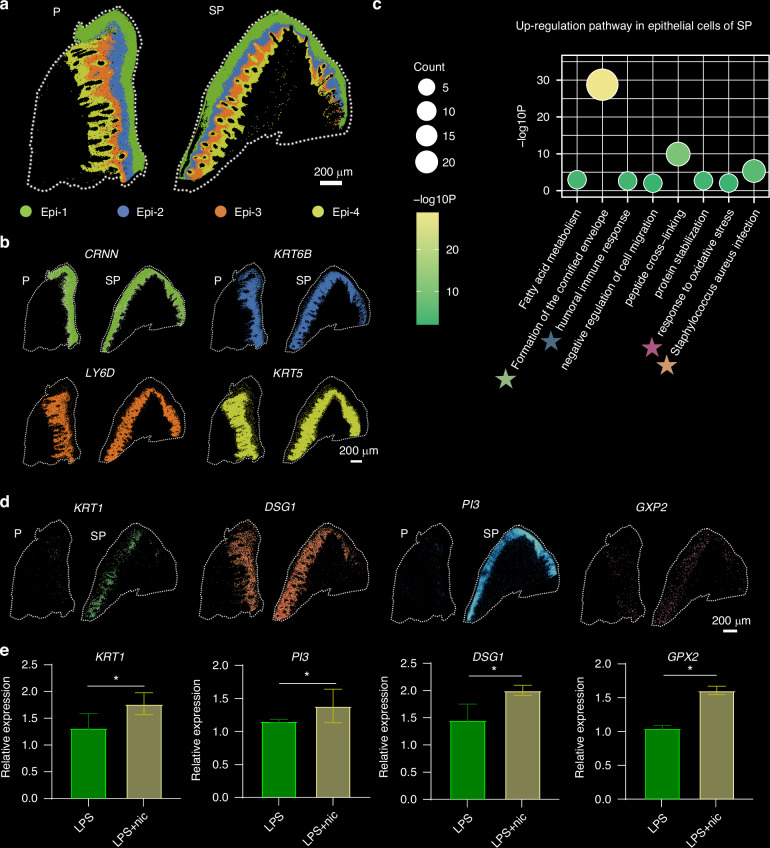
Smoking-induced damage in gingival epithelial cells. **a** Spatial map of the four subgroups of epithelial cells in the Visium HD date. **b** Spatial map of marker gene expression in the four cell subgroups. **c** Dot plots showing upregulated pathways in epithelial cells from the SP group. **d** Visium HD data analysis revealing the expression of *KRT1, DSG1, PI3*, and *GPX2* in the gingival tissue; scale bar = 200 µm. **e** RT–qPCR analysis of the *KRT1*, *DSG1*, *PI3*, and *GPX2* expression levels in epithelial cells treated with LPS and nicotine (*n* = 3). *P < 0.05

Exposure to smoking-related toxins has a direct effect on the integrity of the oral mucosal epithelial barrier, increasing vulnerability to injury. Gingival epithelial cells were classified into four distinct subgroups, and their correlation with tissue morphology was analysed (Fig. 2a). Utilizing clustering data, distinct marker genes were identified for each epithelial subgroup within the gingival tissue morphology: *Cornulin* (*CRNN*) for Epi-1, *Keratin 6B (KRT6B*) for Epi-2, *Ly-6 Domain Containing 1* (*LY6D*) for Epi-3, and *Keratin 5* (*KRT5*) for Epi-4 (Fig. 2b). These genes are essential for maintaining epithelial function and integrity. For instance, *CRNN* is a crucial epithelial cell protein predominantly found in tissues like the skin, oral mucosa, and gastrointestinal tract mucosa, where it has protective, barrier, and repair functions.^18,19^ *KRT6B*, a member of the keratin family, forms network structures with other keratin proteins to uphold cellular mechanical strength and stability, which are particularly vital for barrier function and resilience against external stress.^20,21^ *LY6D*, belonging to the Ly-6 superfamily, encodes a membrane-bound protein involved in cell signalling, emphasizing its role in preserving epithelial homoeostasis.^22,23^ *KRT5*, another keratin family member, is mainly expressed in basal layer cells, where it plays a crucial role in providing structural stability and ensuring epithelial integrity under conditions of friction and stretching.^24,25^ Furthermore, immunofluorescence staining was conducted to validate the spatial localization of these molecular markers in the tissue, confirming consistency with the sequencing results (Supplementary Fig. 2). This validation reinforces the accuracy and biological significance of the single-cell RNA sequencing data and further elucidates the specific expression patterns and functional attributes of these markers in distinct epithelial subgroups.

To investigate the alterations in the gene expression of epithelial cells induced by smoking, we identified DEGs in the gingival epithelial cells of the P and SP groups. Gene Ontology (GO) pathway enrichment analysis revealed that, compared with chronic periodontitis, smoking upregulated the expression of genes associated with pathways such as *Staphylococcus aureus* infection, oxidative stress, the humoral immune response, and keratinized membrane formation in the gingival epithelium (Fig. 2c). Key genes such as *Keratin 1 (KRT1), Desmoglein 1 (DSG 1), Phosphoinositide 3-kinase (PI3)*, and *Glutathione Peroxidase 2 (GPX2)* are crucial genes in the above pathways. These genes are essential for maintaining the structure and function of epithelial cells, connecting epithelial cells, and participating in the stress response and inflammation signalling, as well as the response to oxidative stress. To confirm the spatial expression differences of these pathways in the gingival epithelium, we further mapped these genes in both chronic periodontitis tissues and smoking-associated periodontitis tissues (Fig. 2d), revealing statistically significant differences (Supplementary Fig. 3). Additionally, the in vitro stimulation of gingival epithelial cells with 100 nm LPS mimicked the inflammatory microenvironment, and the combination of LPS and nicotine (100 nm) was used to simulate the effect of smoking on the gingival epithelium. The PCR results confirmed that the changes in the expression of *KRT1*, *DSG1*, *PI3*, and *GPX2* were correlated with the spatial expression patterns observed in the tissue samples (Fig. 2e). The above results indicate that smoking increases the risk of periodontitis by disrupting the structure and function of the gingival epithelium through its impact on the expression and function of these key genes.

### Smoking-induced fibroblast alterations and the dysregulation of fibroblast‒epithelial cell communication

**Fig. 3:**
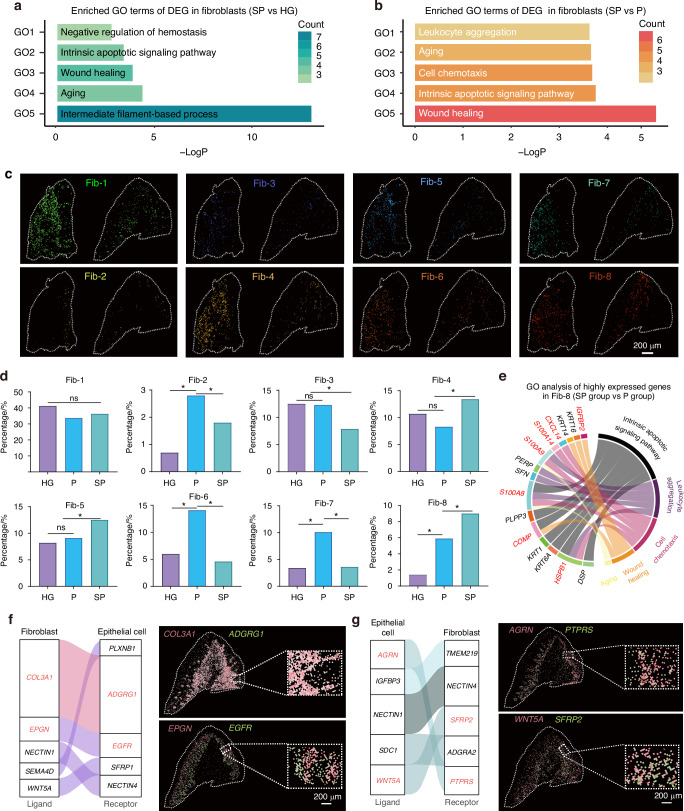
Smoking-induced fibroblast alterations and the dysregulation of fibroblast‒epithelial cell communication. **a** Bar plots showing enriched GO terms of DEGs (SP group vs. HG group) in fibroblasts. **b** Bar plots showing enriched GO terms of DEGs (SP group vs. P group) in fibroblasts. **c** Spatial map of the eight subgroups of fibroblasts in the Visium HD date. **d** Proportions of fibroblasts subgroups in each group. **e** GO analysis of highly expressed genes in Fib-8 (SP group vs. P group). **f** Ligand–receptor interactions between fibroblasts and epithelial cells and Visium HD data showing the cell communication signals of COL3A1-ADGRG1 and EPGN-EGFR. **g** Ligand–receptor interactions between epithelial cells and fibroblasts and Visium HD data showing the cell communication signals of AGRN-PTPRS and WNT5A-SFRP2. *P < 0.05

Gingival fibroblasts play a critical role in maintaining the integrity and function of the gingival epithelium. By conducting GO enrichment analysis, we determined the relevant biological functions of the differentially expressed genes in fibroblasts between the SP and P groups. We specifically focused on statistically significant GO terms that are associated with pathways involving inflammation, immune response, and tissue healing. Our results revealed that individuals in the smoking group, as opposed to healthy controls, presented upregulated expression of genes linked to ageing, intrinsic apoptotic signalling, and mitotic processes (Fig. 3a). In contrast to those associated with chronic periodontitis, genes associated with cell chemotaxis, leucocyte proliferation, and ageing were enriched in the smoking group (Fig. 3b). The subsequent clustering of fibroblasts into eight subgroups allowed mapping of their spatial distribution onto the gingival tissue morphology (Fig. 3c and Supplementary Fig. 4). Statistical assessments of fibroblast subgroup proportions revealed significantly higher Fib-8 expression in the smoking-related periodontitis group than in both the periodontitis group and the normal group (Fig. 3d).

Smoking was found to upregulate the expression of Fib-8 and genes associated with wound healing (e.g., *IGFBP2, COMP*), chemotaxis (e.g., *CXCL14, HSPB1, S100A14*), and ageing (e.g., *S100A8, S100A9*) pathways (Fig. 3e). Analyses of epithelial-fibroblast interactions using CellChat revealed that fibroblasts release ligands that interact with epithelial cell receptors, including COL3A1-ADGRG1, EPGN-EGFR, NECTIN1-NECTIN4, WNT5A-SFRP1, and SEMA4D-PLXNB1. Specifically, the EPGN-EGFR signalling pathway may promote excessive epithelial proliferation, undifferentiated migration, and barrier disruption, ultimately compromising epithelial barrier function (Fig. 3f). An analysis of cell communication between epithelial cells and fibroblasts revealed an increase in signals such as AGRN-PTPRS, IGFBPS-TMEM219, and WNT5A-SFRP2 (Fig. 3g). In smoking-associated chronic inflammatory environments, the upregulation of PTPRS and SFRP2 on fibroblasts aligns with previous studies demonstrating increased expression of these markers in fibroblasts with a pro-inflammatory phenotype.^26,27^ The accumulation of pro-inflammatory fibroblasts may drive abnormal epithelial cell proliferation,^28^ compromise epithelial barrier stability, and facilitate the infiltration of bacteria or inflammatory factors, thereby exacerbating the inflammatory response.^29,30^ Signals such as AGRN-PTPRS and WNT5A-SFRP2 may lead to fibroblast dysfunction or abnormal proliferation, thereby exacerbating damage to periodontal tissues.^27,31–36^ These results suggest that smoking upregulates the expression of genes related to ageing, chemotaxis, and wound healing in gingival fibroblasts and that the dysregulation of epithelial‒fibroblast interactions potentially affects the integrity and function of periodontal tissues.

### Smoking induces immune microenvironment disruption by promoting macrophage dysfunction

**Fig. 4:**
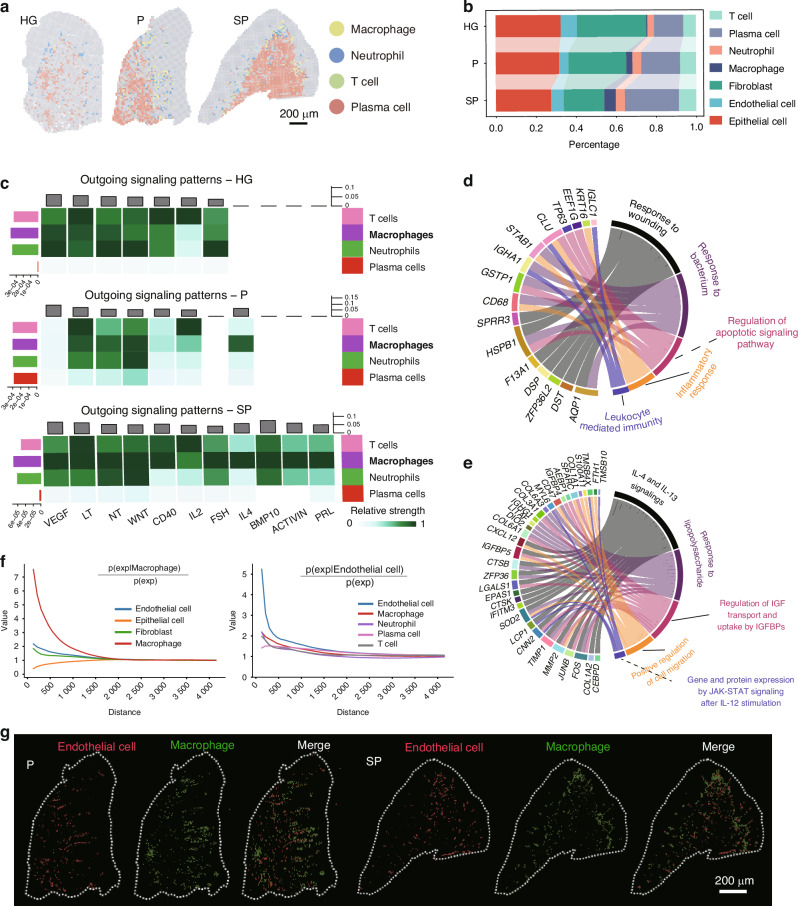
Smoking induces an immune microenvironment disruption by promoting macrophage dysfunction. **a** Map showing the infiltration of the four immune cell subgroups into the gingiva. **b** Proportions of each immune cell subgroup in each group. **c** CellChat analysis revealing outgoing signalling patterns of each immune cell subgroup in each group. **d** GO analysis of highly expressed genes in macrophages (SP group vs. P group). **e** GO analysis of downregulated genes in macrophages (SP group vs. P group). **f** Spatial analysis revealing the spatial relationship of macrophages and endothelial cells. **g** Visium HD data showing the spatial map of macrophages and endothelial cells

Smoking disrupts the local immune microenvironment in periodontal tissues. To investigate its impact on each subgroup, immune cells in gingival tissue were categorized into four subpopulations: plasma cells, macrophages, T cells, and neutrophils (Fig. 4a). Analyses revealed a significant increase in immune cell numbers in smoking-related periodontitis tissues (40.6% of total cells), with plasma cells exhibiting the most notable increase from 18.7% in chronic periodontitis to 27.8% in smoking-related periodontitis. The percentage of macrophages also increased from 3% in chronic periodontitis to 5.6% in smoking-related periodontitis (Fig. 4b).

The CellChat analysis of signalling patterns indicated that macrophages in the smoking group significantly upregulated the expression of genes in proinflammation-related signalling pathways, such as *VEGF, WNT, CD40*, and *IL2* (Fig. 4c). Differential gene analysis of macrophages revealed that, compared with those in the chronic periodontitis group, the expression of genes related to the “response to wounding,” “leucocyte-mediated immunity,” “regulation of apoptotic signalling,” “response to bacteria,” and “inflammatory response” pathways was upregulated in the smoking group (Fig. 4d). The downregulated genes were associated with the following pathways: “interleukin-4 and interleukin-13 signalling,” “positive regulation of cell migration,” and “regulation of insulin-like growth factor transport” (Fig. 4e).

Utilizing Squidpy’s co-occurrence score, we analysed the Visium dataset and identified significant spatial interactions among distinct cell clusters. In particular, macrophages displayed closer spatial proximity to endothelial cells than to other cell types, such as neutrophils, T cells, and plasma cells (Fig. 4f). Furthermore, in the comparison of distances between endothelial cells and macrophages across the three groups, the SP group exhibited the shortest proximity between these cell types (Supplementary Fig. 5). Spatial mapping of macrophages and endothelial cells in the gingiva section illustrated that macrophages were predominantly situated around endothelial cells, particularly in the smoking group (Fig. 4g). The intimate spatial association between macrophages and endothelial cells implies a potentially important role of their interaction in the progression of SP. Collectively, these findings indicate the significant impact of smoking on perturbing the immune microenvironment in periodontal tissues through the modulation of macrophage function, augmentation of inflammatory signalling, and alteration of gene expression, consequently exacerbating tissue damage.

### Endothelial cell inflammation and its interaction with macrophages potentially aggravate SP

**Fig. 5:**
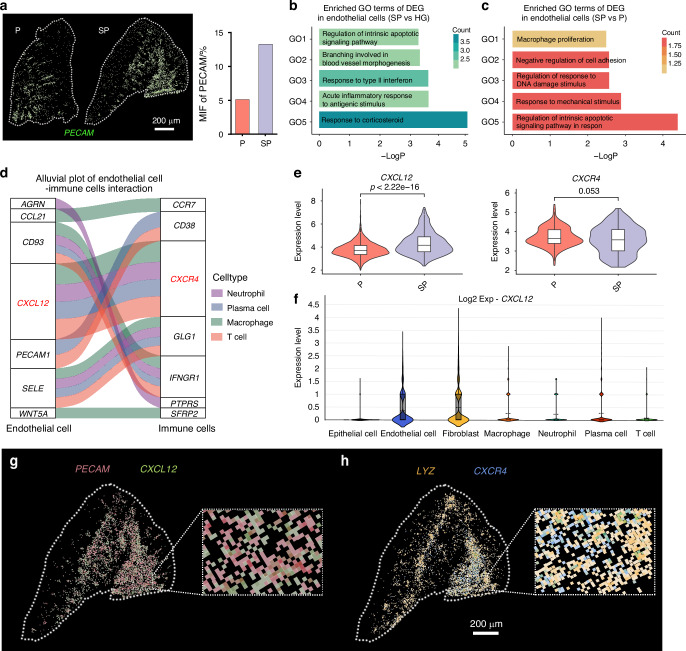
Endothelial cell damage in the gingiva is an important factor contributing to the immune microenvironment disruption. **a** Visium HD data analysis revealing the relative expression levels of *PECAM* (marker gene of endothelial cells). **b** Bar plots showing enriched GO terms of DEGs (SP group vs. HG group) in endothelial cells. **c** Bar plots showing enriched GO terms of DEGs (SP group vs. P group) in endothelial cells. **d** Alluvial plot showing the interaction of endothelial cells–immune cells. **e** Visium HD data analysis revealing the relative expression levels of *CXCL12* and *CXCR4* in the gingival tissue. **f** Violin plot showing the relative expression levels of *CXCL12* in each cell subgroup. **g** Visium HD data analysis revealing the colocalization of *PECAM* ^*+*^ cells and *CXCL12*^*+*^ cells. **h** Visium HD data analysis revealing the colocalization of *LYZ*^*+*^ cells and *CXCR4*^*+*^ cells

We first focused on functional changes in endothelial cells in the gingival tissue of SG to investigate the role of the interaction between endothelial cells and macrophages in exacerbating periodontal inflammation. *PECAM*, a marker of endothelial cells, showed increased expression in the SP group compared to the P group (Fig. 5a). We then examined gene expression in endothelial cells across the HG, P, and SG groups. Compared to HG individuals, SP patients exhibited a significant upregulation of genes associated with DNA damage, intrinsic apoptotic signalling, vascular morphogenesis, and acute inflammatory responses to antigen stimulation (Fig. 5b). Moreover, the TF‒gene network analysis identified downstream gene targets of key transcription factors such as NFKB1, RELA, and STAT3, suggesting their involvement in the observed differential gene expression in the endothelial cells of the smoking group (Supplementary Fig. 6a).

Differences in gene expression in endothelial cells between the SP group and P group were analysed. GO analysis revealed that smoking-associated periodontitis upregulated the expression of genes involved in DNA damage regulation, apoptosis signalling, macrophage proliferation, and responses to DNA damage and bacterial stimulation (Fig. 5c). The activation of DNA damage and apoptosis pathways in endothelial cells could impair their function and compromise vascular endothelial integrity,^37^ fostering an inflammatory milieu and augmenting the recruitment and activation of immune cells, particularly macrophages, thereby exacerbating periodontal tissue degradation.^38^ Additionally, compromised endothelial cell responses to bacterial stimulation may intensify local inflammation, thereby aggravating periodontitis in smokers.^39^ An analysis of transcription factors revealed the participation of inflammatory-related factors, such as NFKB1, SP1, RELA, STAT3, and HIF1A, in the endothelial cells of the SP (Supplementary Fig. 6b). Therefore, endothelial cell dysfunction likely exacerbates periodontal inflammation in the context of smoking.

Furthermore, our analysis of cell communication between endothelial and immune cells in individuals with smoking-related periodontitis conditions reveals that endothelial cell-derived CXCL12 influences macrophages by binding to CXCR4. The expression level of *CXCL12* was higher than that of other endothelial cell ligands binding to macrophage receptors (Fig. 5d). A comparison of *CXCL12* expression in endothelial cells between the P and SP groups confirmed the significant upregulation of *CXCL12* in endothelial cells of the SP group (Fig. 5e). Additionally, endothelial cells were identified as the primary source of *CXCL12* (Fig. 5f). Spatial transcriptomic visualization further supported the activation of *CXCL12*-*CXCR4* signalling between endothelial cells and macrophages in the SG group, showing spatial proximity between endothelial cells with high *CXCL12* expression (Fig. 5g) and macrophages with high *CXCR4* expression (Fig. 5h). Taken together, endothelial cell inflammation and its interaction with macrophages may exacerbate SP.

### Targeting Endothelial *CXCL12* Promotes Macrophage Polarization to an Anti-Inflammatory Phenotype, Alleviating Periodontal Inflammation and Bone Resorption

**Fig. 6:**
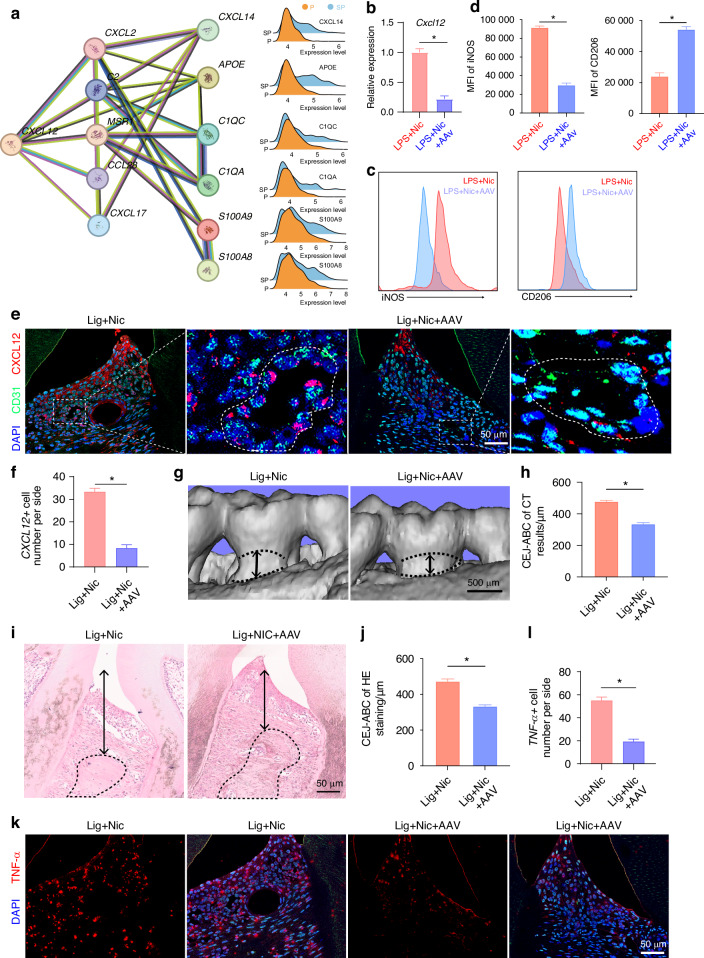
Targeting endothelial *CXCL12* promotes macrophage polarization to an anti-inflammatory phenotype, alleviating periodontal inflammation and bone resorption. **a** Associations between endothelial CXCL12 signalling and macrophage inflammation-related genes were predicted using the STRING database. **b** RT–qPCR analysis of the *CXCL12* expression levels in endothelial cells treated with AAV-shRNA-*CXCL12*. **c**, **d** Flow cytometry analysis showing the expression of iNOS and CD206 in macrophages treated with different endothelial cell culture media. **e**, **f** Multicolour IF staining showing the expression of CXCL12 and the endothelial cell marker CD31. The nuclei were stained with DAPI. Bar = 50 μm. **g**, **h** Three-dimensional reconstructions of the maxilla in each group generated by micro-CT scans; the black arrows indicate the distance between cement enamel junction and alveolar bone crest (CEJ-ABC), scale bar = 500 μm. **i**, **j** Images of the HE-stained periodontium from each group showing bone resorption; the black arrows indicate the distance between CEJ-ABC, scale bar = 50 μm. **k**, **l** IF staining showing the expression of TNF-α. Bar = 50 μm. *P < 0.05

Given the higher *CXCL12* expression in endothelial cells from the SP group compared to the P group and the proximity of endothelial cells to macrophages, we investigated CXCL12-regulated genes in macrophages. The prediction using the STRING database suggests that CXCL12 may upregulate proinflammatory genes, such as *CXCL14, S100A9*, and *S100A8*. Consistent with this result, our spatial transcriptomic data showed upregulated expression of *CXCL14, APOE, C1QC, C1QA, S100A9*, and *S100A8* in macrophages of the SG group compared to the P group (Fig. 6a).

Furthermore, we explored the impact of endothelial cell-derived CXCL12 on macrophage functions. Tie1 is an endothelial cell-specific promoter; we constructed AAV-Tie1-shRNA*-CXCL12* (AAV-sh*CXCL12*) to drive the expression of an shRNA via the Tie1 promoter and specifically inhibit the expression of *CXCL12* in endothelial cells. We stimulated endothelial cells with nicotine while simultaneously introducing AAV-sh*CXCL12* and control AAV (AAV-con), and the RT-qPCR analysis revealed that the expression of *CXCL12* in endothelial cells was significantly downregulated after the addition of AAV-sh*CXCL12* (Fig. 6b). The collected endothelial cell culture medium was then added to bone marrow-derived macrophages (BMDMs). The medium from endothelial cells with suppressed *CXCL12* expression reduced the proinflammatory shift in macrophages, with the downregulation of the proinflammatory-related gene *CD86* and upregulation of the anti-inflammatory-related gene *CD206* (Fig. 6c, d).

Furthermore, in a mouse model of smoking-induced periodontal inflammation simulated by ligature placement and nicotine injection, immunofluorescence staining revealed that treatment with AAV-sh*CXCL12* targeting endothelial cells led to a marked downregulation of *CXCL12* expression in endothelial cells (Fig. 6e, f). Micro-CT analysis revealed that treatment with AAV-sh*CXCL12* alleviated the damage to the alveolar bone caused by local nicotine injection (Fig. 6g, h). HE staining revealed that alveolar bone destruction in mice was significantly relieved following AAV-sh*CXCL12* treatment (Fig. 6i, j). Moreover, the immunofluorescence results revealed a substantial reduction in the expression of inflammatory cytokines, such as TNF-α, within the periodontal tissues (Fig. 6k, l). The interaction between macrophages and endothelial cells, which is mediated by *CXCL12* signalling, exacerbates smoking-induced periodontal inflammation, and targeting *CXCL12* in endothelial cells with AAV-sh*CXCL12* can reduce proinflammatory macrophage activation and alleviate alveolar bone destruction in a mouse model.

## Discussion

Using single-cell spatial transcriptomics, this study revealed that smoking significantly alters the cellular landscape, immune microenvironment, and cellular interactions within periodontal tissues, driving periodontitis progression. Notably, targeting endothelial CXCL12 promotes macrophage polarization towards the M2 anti-inflammatory phenotype, mitigating periodontal inflammation and bone resorption. These findings offer new insights into the pathogenesis of smoking-associated periodontitis and highlight potential therapeutic targets.

Compared to previous single-cell sequencing studies of periodontitis,^40^ our research has several unique features. We employed Visium HD single-cell spatial transcriptomics, a high-resolution platform that enables gene expression analysis at single-cell resolution while preserving spatial information within tissue samples. This approach offers an advantage over other single-cell RNA sequencing methods by capturing the spatial context of cellular interactions. Unlike previous studies that primarily focused on immune cell populations, our research emphasizes the complex interactions between multiple cell types, particularly vascular cell–immune cell crosstalk, providing deeper insights into how these interactions contribute to disease progression, especially in smoking-induced periodontitis. A key strength of our study is the use of formalin-fixed paraffin-embedded (FFPE) tissue samples, which do not require fresh-frozen tissue.^14^ This approach allows us to fully utilize biobank resources and analyse archived clinical samples. This advantage is especially valuable for studying chronic conditions like periodontitis, where cell–cell interactions play a critical role in disease development. These features highlight the benefits of using spatial transcriptomics with FFPE tissue samples to comprehensively explore cellular dynamics and tissue interactions in periodontitis.

We observed increased expression of keratin-related genes, such as *KRT1* and *KRT5*, which are linked to epithelial remodelling under stress.^41,42^ The gene expression profile in smoking-associated periodontitis suggests a pathological shift towards a hyperproliferative and inflammatory epithelial phenotype, likely disrupting mucosal barrier function in periodontal tissues.^10,43^ DSG1 plays a crucial role in maintaining the integrity and stability of the gingival epithelium.^44^ Smoking may upregulate *DSG1* expression, contributing to the breakdown of cell‒cell junctions and increasing the sensitivity of gingival tissue to external stimuli, thereby promoting periodontitis development.^45,46^ In gingival epithelial cells, PI3K (phosphoinositide 3-kinase) activation is closely associated with stress and inflammatory responses.^47^ Smoking may enhance PI3K signalling, exacerbating the inflammatory response in gingival cells and aggravating the pathological process of periodontitis.^48^ Additionally, smoking suppresses the expression of the antioxidant enzyme *GPX2*, reducing the antioxidant enzyme GPX2 and reducing the antioxidant capacity of gingival epithelial cells.^49^ This change may lead to increased cellular damage and inflammation, further contributing to the onset and progression of periodontitis.^50^

Fibroblasts play a critical role in maintaining the homoeostasis of gingival tissue,^51^ and our analysis highlights significant alterations in fibroblast gene expression following smoking. The increased expression of Fib-8 in smoking-related periodontitis also suggests a shift in fibroblast subpopulation dynamics.^52^ Notably, the smoking-induced upregulation of genes associated with ageing, apoptosis, and cell migration, such as *S100A8* and *S100A9*, aligns with earlier findings that smoking promotes fibroblast dysfunction and impaired wound healing.^53,54^ The expression of genes involved in wound healing pathways in the SP group was elevated, primarily due to the ongoing damage and chronic inflammatory response induced by smoking. In response to damage, the body activates repair mechanisms that increase the expression of wound healing-related genes, whose upregulation subsequently promotes cell proliferation and migration.^51^ However, prolonged and excessive repair may lead to tissue dysfunction, further exacerbating the progression of periodontitis.^55^ Furthermore, the communication between fibroblasts and epithelial cells, particularly through the EPGN-EGFR signalling pathway, may drive excessive epithelial proliferation and migration,^56,57^ which could contribute to the epithelial barrier breakdown observed in smokers. These fibroblast‒epithelial interactions, if not properly regulated, may exacerbate the inflammatory response in periodontal tissues.^14,58^

Our study identified smoking-induced changes in immune cell composition and function within the gingival tissue. Specifically, we observed an increased number of plasma cells and macrophages in the SP group, a finding that is consistent with previous reports linking smoking to increased immune cell recruitment and activation in periodontal tissues.^3,11^ Gene enrichment analysis of immune cells revealed the significant activation of pathways associated with inflammation, immune responses, and bacterial infection, including the response to wounding and leucocyte-mediated immunity. The increase in macrophages and their activated inflammatory pathways suggests that smoking may induce macrophage dysfunction, leading to an exaggerated inflammatory response and potentially contributing to tissue destruction.^59^ This finding is further supported by our spatial analysis, which revealed that macrophages in smoking-related periodontitis are clustered around blood vessels and possibly interact with endothelial cells to amplify local inflammation.^60^

A key novel finding of this study is the significant damage to endothelial cells in smoking-associated periodontitis. Reduced endothelial cell density and dysregulated gene expression related to DNA damage, apoptosis, and inflammation underscore the pivotal role of endothelial dysfunction in exacerbating periodontal inflammation. The upregulation of the expression of genes such as *VWF* and *PECAM* in endothelial cells indicates potential alterations in vascular integrity and blood flow, contributing to the compromised immune response in the gingiva.^61,62^ Our transcription factor analysis further highlights the involvement of NFKB1, STAT3, and RELA in the observed endothelial dysfunction.^63–65^ These transcription factors are central to inflammatory responses and could represent potential therapeutic targets for mitigating smoking-induced periodontal tissue damage.^66,67^ Additionally, the interaction between macrophages and endothelial cells, facilitated by key signalling pathways, may create a feedback loop that perpetuates inflammation and tissue destruction.^68^

Our findings suggest that smoking-induced endothelial dysfunction and macrophage activation are central to the disruption of the periodontal immune microenvironment. Therefore, we constructed an AAV-shRNA-Cxcl12 targeting endothelial cells. In vivo experiments using an AAV-mediated reduction in *Cxcl12* in endothelial cells provided promising results, revealing a shift in macrophage polarization towards the M2 anti-inflammatory phenotype and a reduction in periodontal inflammation. Then, we attempted to use this endothelial cell-targeted AAV-shRNA-Cxcl12 to determine whether it could alleviate endothelial–immune microenvironment disorders caused by double injury from ligation and nicotine. The results also confirmed that regulating the abnormal *Cxcl12* signalling in endothelial cells could alleviate local periodontal inflammation and reduce periodontal bone destruction. These findings highlight the potential of targeting endothelial‒macrophage interactions or specific inflammatory pathways as therapeutic strategies for treating smoking-associated periodontitis.

Targeting CXCL12 shows promise in mitigating inflammation and bone resorption in individuals with smoking-induced periodontitis. However, the dual role of CXCL12 in immune responses and tissue repair must be considered, as it governs immune cell migration and activation, which are crucial for wound healing and tissue regeneration.^69^ Excessive CXCL12 inhibition may disrupt normal tissue repair and compromise the self-repair capacity of periodontal tissues. Therefore, future strategies targeting CXCL12 need to carefully balance its therapeutic effects with its role in tissue repair. Future research should investigate local delivery systems, like nanoparticles or liposomes,^70,71^ to reduce systemic side effects and improve treatment precision.

Furthermore, while some genes were validated in this study, further exploration of other pertinent genes and pathways is necessary to fully comprehend the molecular mechanisms involved in smoking-induced periodontitis. The study also underscores the importance of examining other smoking constituents, such as tar, which could impact periodontal tissue responses. This study focused on the therapeutic effects of AAV-sh*CXCL*12 on the SP phenotype. Subsequent studies should assess its effects on non-smoking periodontitis to bolster the findings and provide a more comprehensive insight into its therapeutic potential. In addition, gingiva samples from flap surgery are predominantly composed of connective tissues with minimal epithelial presence, while tissue specimens from crown lengthening procedures contain both epithelial and connective tissues. Discrepancies in tissue composition between the two groups may complicate the interpretation of results.

In summary, smoking-induced alterations in gingival epithelial, fibroblast, immune, and endothelial cell populations significantly contribute to the pathogenesis of periodontal disease. Our study reveals the complex interactions between these cellular components, which collectively disrupt the local immune microenvironment and promote tissue destruction. Future therapeutic strategies targeting these pathways, particularly endothelial dysfunction and macrophage polarization, may offer new avenues for treating smoking-related periodontal diseases.

## Methods and Materials

### Tissue collection and preparation

The periodontal tissue samples used in this study were obtained from 8 patients with periodontitis (KQEC-2024-30-01), including four with chronic periodontitis (P), four with smoking-associated periodontitis (SP), and four healthy controls (HCs). All participants provided informed consent, and the study was conducted in accordance with ethical guidelines. The tissue samples were surgically collected and immediately fixed in 10% formalin. The samples were subsequently processed using the formalin‒fixed paraffin‒embedded (FFPE) method and embedded in tissue blocks. The FFPE tissue blocks were sectioned and prepared for spatial transcriptomics analysis using the Visium HD platform.

### Patient enrolment and tissue collection methods

#### Inclusion Criteria

##### Chronic Periodontitis (P)

Patients were diagnosed according to the 2018 periodontal disease classification with Stage III or IV periodontitis (clinical attachment loss >5 mm; PD > 6 mm).

##### Smoking-Associated Periodontitis (SP)

Patients met the same criteria as those with chronic periodontitis but had a smoking history of >5 years and consumed >10 cigarettes per day.

##### Healthy Controls (HCs)

These individuals had no systemic diseases, no periodontal treatment in the past 6 months, and no signs or symptoms of soft tissue disease, oral or dental infections, or mild gingivitis.

#### Exclusion Criteria

##### Systemic Diseases

Presence of systemic conditions such as diabetes, autoimmune diseases, malignancies, or pregnancy or lactation.

##### Recent Medications

Use of systemic antibiotics, systemic or inhaled corticosteroids, immunosuppressive agents, chemotherapy, or high-dose commercial probiotics within 3 months prior to enrolment.

##### Tobacco Use

Use of tobacco products, including e-cigarettes, within 1 year prior to enrolment.

#### Tissue collection methods

Healthy tissue samples were collected during crown lengthening procedures from gingival tissues with no radiographic evidence of bone loss and probing depths ≤4 mm.

Diseased tissue samples were collected during flap surgeries from sites with radiographic evidence of bone loss and probing depths >5 mm.

The submarginal incision positioned approximately 1–3 mm from the gingival margin, extending above the alveolar bone crest.

### RNA quality assessment and FFPE sample preparation

Additional sections (4–5 sections, approximately 5 μm thick) were cut from each FFPE tissue block for the RNA quality assessment to ensure the quality of the RNA for the spatial transcriptomic analysis. The RNA quality was evaluated based on the DV200 metric (Agilent 5300 Fragment Analyzer System), which measures the percentage of RNA fragments longer than 200 base pairs (bp). Samples with a DV200 value greater than 50% were considered qualified for further experimentation. Notably, while the Visium CytAssist platform requires a minimum DV200 threshold of 30%, our study adopted a more stringent criterion (DV200 > 50%) to ensure higher RNA integrity and the reliability of the downstream spatial transcriptomic data. This rigorous quality control step was essential for generating accurate and meaningful results in the subsequent analyses.

### Single-cell RNA transcriptomics analysis

To investigate the gene expression patterns of various cell populations in the spatial transcriptomics data, we first performed single-cell RNA (scRNA) transcriptomic analysis. The data were initially imported into R and filtered on the basis of the following criteria: (1) nCount_RNA < 20 000; (2) nFeature_RNA < 5 000; and (3) mitochondrial gene content < 15%. After filtration, all the samples were pooled. Normalization, followed by principal component analysis (PCA) for dimensionality reduction, was performed using the Seurat package (v.4.4.0). The Harmony package (v.1.2.0) was used for batch correction between samples using the first 50 principle components produced via principal component analysis. Then, we identified clusters using the Seurat function FindClusters (clustering resolution = 0.5). All cell types were defined on the basis of the expression of marker genes. Finally, uniform manifold approximation and projection (UMAP) was utilized for the visualization of cell clusters in reduced dimensions.

### Spatial transcriptomic analysis

Spatial transcriptomics was performed using the Visium HD platform from 10x Genomics. The capture area of the Visium HD platform is typically 6.5 × 6.5 mm^2^. Each capture area is subdivided into millions of 2 × 2 μm^2^ microgrids, with a unique barcode for each one, allowing the capture and labelling of RNA within the tissue. The captured RNA is then amplified and labelled to generate libraries for downstream sequencing analysis. The RNA sequences are mapped to a reference genome, with low-quality reads removed, to obtain gene expression levels and spatial distribution information for each capture area.

The spatial transcriptomics data were imported into R. Data normalization and principal component analysis (PCA) for dimensionality reduction were performed as described above. We then integrated cell type annotations and gene expression profiles from the scRNA dataset to examine the proper distribution of these cell types in the spatial transcriptomics data. On the basis of this information, we successfully clustered all the cells in the spatial transcriptomic data and used UMAP to visualize the cell clusters. The marker genes of each cell cluster were calculated and are presented as dot plots.

### Differential expression and pathway analysis

Differential gene expression analysis between different cell clusters or experimental groups was conducted using the Seurat package. Differentially expressed genes (DEGs) were filtered using the following criteria: 1) *P* value < 0.05 and 2) logFC > 0.5. The expression of genes across different groups was visualized using dot plots.

Gene Ontology (GO) enrichment analysis was performed using the clusterProfiler package (v.4.8.3) to examine the enriched pathways associated with DEGs. This analysis provides insights into the differences in biological behaviour between different groups. The GO enrichment results were filtered by *P* value < 0.05, and the top enriched pathways are displayed in bar plots.

### Cell‒cell communication analysis

The CellChat package (v.1.6.1) was used to analyse cell‒cell communication, focusing on ligand‒receptor interactions between different cell clusters and their spatial distribution within the tissue. The CellChat package identifies the ligands and receptors expressed in each cell cluster and predicts potential interactions between them, constructing a network of intercellular communication.

Further analysis of these interactions allowed us to identify active cell signalling pathways and assess the strength of outgoing signalling pathways across different cell types. The results were visualized as heatmaps, highlighting the most prominent signalling pathways. We subsequently examined the expression intensity of the respective ligand‒receptor pairs in the spatial transcriptomics data, which were used to construct a spatial communication map.

#### Transcription factor analysis

In this study, transcription factor (TF) analysis of DEGs was conducted using the NetworkAnalyst web platform (https://www.networkanalyst.ca/) and its integrated JARSTAT database. After DEGs from the RNA-seq data were identified, those genes were input into the JARSTAT module of NetworkAnalyst to identify potential transcription factors that might regulate them. The tool utilizes known TF‒gene interaction data to predict binding motifs within the promoter regions of DEGs. Enrichment analysis was performed to identify TFs significantly associated with the DEGs, with statistical significance assessed using hypergeometric tests. The results were visualized through TF-target gene networks, highlighting key transcription factors and their potential regulatory roles in the biological processes underlying the differential expression.

### Cell culture

#### Gingival epithelial cells

Gingival tissues with keratinized epithelium were obtained from patients under 30 years of age during crown lengthening or the extraction of fully impacted teeth. The tissues were transferred to the laboratory, cleaned thoroughly, and immersed in culture medium containing 10 mg/mL dispase II (type II dispase) for 8 hours at 4 °C. Gingival epithelial cells were microscopically separated, minced, and digested at 37 °C for 10 minutes. The digestion was terminated by adding complete culture medium supplemented with FBS, and the cells were cultured in oral keratinocyte-specific medium at 37 °C in a 5% CO₂ incubator. Experiments were conducted using cells from passages 3–5. The cells were stimulated for 24 hours with 100 ng/mL lipopolysaccharide (LPS) and 100 ng/mL LPS combined with 100 nmol/L nicotine. After stimulation, the cell samples were collected for further analysis.

#### Mouse Endothelial Cells (MECs)

Mouse endothelial cells (MECs) were purchased from Wuhan SUNNCELL Biotechnology Co., Ltd. (Cat NO: SNP-M120). After thawing, the cells were seeded into T25 culture flasks and cultured at 37 °C in a 5% CO₂ humidified incubator. The culture medium was changed every 48–72 hours, and subculturing was performed when the cell monolayer reached 80%–90% confluence. The cells used for the experiments were from passages 4–8 to ensure phenotypic stability. To interfere with the Cxcl12 gene in MECs, AAV transfection was performed. The cells were stimulated with 100 ng/mL LPS and 100 nmol/L nicotine for 24 hours, and Cxcl12 gene expression was compared between the transfected and nontransfected MECs. After the stimulus was removed, the supernatants from the cell cultures were collected for analysis at 6 hours.

#### Mouse Bone Marrow-Derived Macrophages (BMDMs)

For the isolation of BMDMs, 4-week-old mice were euthanized, and the femurs and tibias were dissected. The bones were washed with PBS, and the epiphyseal ends were trimmed using scissors. The bone marrow was flushed with RPMI-1640 medium, resuspended in medium containing 10% FBS and 1% penicillin/streptomycin, and plated in culture dishes. L929 cell-conditioned medium was added to the culture at a 20% concentration, and the cultures were maintained at 37 °C in a 5% CO₂ incubator. The medium was changed every 2–3 days, and after 1 week, the cells were induced to differentiate into macrophages. Following 24 hours of coculture with endothelial cell-conditioned medium, the macrophage phenotype was assessed using flow cytometry.

#### Real-Time Quantitative PCR (qPCR)

Total RNA was extracted from cultured gingival epithelial cells and mouse endothelial cells using NucleoZOL reagent (MACHEREY‑NAGEL). Complementary DNA (cDNA) was synthesized using a PrimeScript RT Reagent Kit (TaKaRa). Gene expression levels were measured by qPCR using SYBR PCR Master Mix (Roche, Indianapolis, IN, USA) in a QuantStudio™ 7 Flex System. The sequences of the PCR primers used in this study are listed in Supplementary Table S1.

#### Flow Cytometry

BMDMs were collected, centrifuged, washed, and then resuspended in 100 µL of PBS at a concentration of 1 × 10⁶ cells per tube. The cells were incubated on ice for 30 minutes with a PE-conjugated anti-mouse iNOS antibody in the dark. For intracellular CD206 staining, the cells were fixed with 0.5 mL of fixation buffer (BioLegend) for 20 minutes in the dark after surface staining, followed by centrifugation at 350×*g* for 5 minutes according to the manufacturer’s protocol. An APC-conjugated anti-mouse CD206 antibody was added, and the cells were incubated at 4 °C for 20–30 minutes in the dark. After washing, the cells were resuspended and analysed using flow cytometry (CytoFLEX, Beckman Coulter). At least three independent experiments were conducted for each analysis. The data were analysed using FlowJo V10.0 software (Tree Star, Ashland, OR, USA).

#### Animals and Micro-CT

Eight-week-old male C57BL/6 J mice were purchased from the Laboratory Animal Centre of Sun Yat-sen University. All experiments were approved by the Institutional Animal Care and Use Committee (IACUC) of Sun Yat-sen University (SYSU-IACUC-2024-002561).

A mouse periodontitis model was established as described in previous studies. In brief, the mice were placed in a sealed container with 4% isoflurane vapour flow until they were fully anaesthetized. A 5–0 silk suture was tied around the left second maxillary molar to induce ligature-induced periodontitis, and the ligature was retained for 14 days. In parallel, nicotine was dissolved in physiological saline and administered at a dose of 1 mg/kg nicotine via local injection into the periodontal tissue of the maxillary molars in mice three times per week for two weeks to simulate smoking-induced periodontitis. For *Cxcl12* gene silencing, a recombinant adeno-associated virus (AAV) vector was used, where the Tie1 promoter (an endothelial cell-specific promoter) drove the expression of the mouse *Cxcl12* shRNA. The AAV vector (titre: 1×10¹² GC/mL) containing the *Cxcl12* gene silencing construct or an equal volume of control AAV was injected into multiple sites within the local periodontal tissue. Each injection delivered 4 μL of the viral suspension, and was administered twice weekly. After ligature removal, the treatment was continued for one more week, and samples were collected for analysis one week after the final injection.

For micro-CT analysis, the maxillae of experimental mice were collected, fixed in 4% PFA for 24 hours, and washed three times with PBS. The samples were dehydrated in 75% ethanol and placed in standardized cylindrical sample holders. High-resolution micro-CT scanning was performed (Scano Medical AG, Bassersdorf, Switzerland) with the following parameters: 70 kV, 114 mA, 20 µm resolution, and 3 000 millisecond exposure time. Image reconstruction and 3D analysis were conducted using VGStudio MAX 1.2.1 software (Heidelberg, Germany). The distance between the cementoenamel junction (CEJ) and alveolar bone crest (ABC) was measured at six predefined sites on the buccal and palatal sides as described previously.

#### Histology and Immunofluorescence (IF) Staining

For histological analysis, the maxillae of experimental mice were fixed in 4% PFA and decalcified in 0.5 mol/L EDTA for two weeks. After dehydration and paraffin embedding, tissue sections were prepared. Haematoxylin and eosin (H&E) staining was performed, and the CEJ-ABC distances were measured to assess bone loss.

For IF staining, paraffin sections of mouse maxillae were deparaffinized and rehydrated, followed by antigen retrieval. The sections were incubated overnight at 4 °C with primary antibodies and then at room temperature for 60 minutes with appropriate secondary antibodies. The sections were counterstained with DAPI for 3 minutes at room temperature. IF signals were visualized and recorded using an LSM780 laser scanning confocal microscope (Zeiss, Germany).

#### Statistical analysis

All cell and animal experimental data were analysed using GraphPad Prism software. The quantitative data are expressed as means ± standard deviations (means ± SDs). Differences between groups were assessed using one-way ANOVA or t tests, and a *P* value of less than 0.05 was considered statistically significant.

All the statistical analyses were performed using R software. The significance of the data was assessed using the Wilcoxon test, with a *P*-value of less than 0.05 considered statistically significant. The results were visualized primarily using the ggplot2 package (v.3.5.0).

## Supplementary information

Supplemental materials

## Data Availability

The data that support the findings of this study has been uploaded to the National Genomics Data Center (https://ngdc.cncb.ac.cn/omix/releaseList) under accession codes OMlX010737. The published datasets used in this study are available under the following accession codes: GSE164241 from GEO (human scRNA-seq). This paper does not report the original code. The authors will supply the relevant data in response to reasonable requests.
