# Supplementary material for: Single-cell spatial atlas of smoking-induced changes in human gingival tissues
Source: Int J Oral Sci. 2025 Aug 1;17:60. doi: 10.1038/s41368-025-00385-5 (PMC12317052; doi:10.1038/s41368-025-00385-5)
Supplement: Supplementary file 1 — Supplemental materials [file 41368_2025_385_MOESM1_ESM.pdf]

# Supplemental Information

Table 1 Primers used in this study

| GENE | PRIMER SEQUENCE (5'-3') |
|---|---|
| *KRT1* | AAGGAGAGTGGACCAACTGA |
| | AAGCACCATCCACATCCTTC |
| *PI3* | GTTCCCCAGTGAGAGGGA |
| | TGGGAGGAAGAATGGACAGT |
| *DSG1* | ACCCAATCGCCAAAATTCAC |
| | TTGGCCCATTGAGTTCAGAG |
| *GPX2* | CTGGATGGGGAGAAGGTAGAT |
| | ATTCTGACAGTTCTCCTGATGTC |
| *GAPDH* | AGATCCCTCCAAAATCAAGTGG |
| | GGCAGAGATGATGACCCTTTT |
| *Cxcl12* | CACTTGCCAAGCTCCAACTT |
| | ACCCAGCTAAAGGTCCTCAC |
| *Gapdh* | AGGTCGGTGTGAACGGATTTG |
| | TGTAGACCATGTAGTTGAGGTCA |

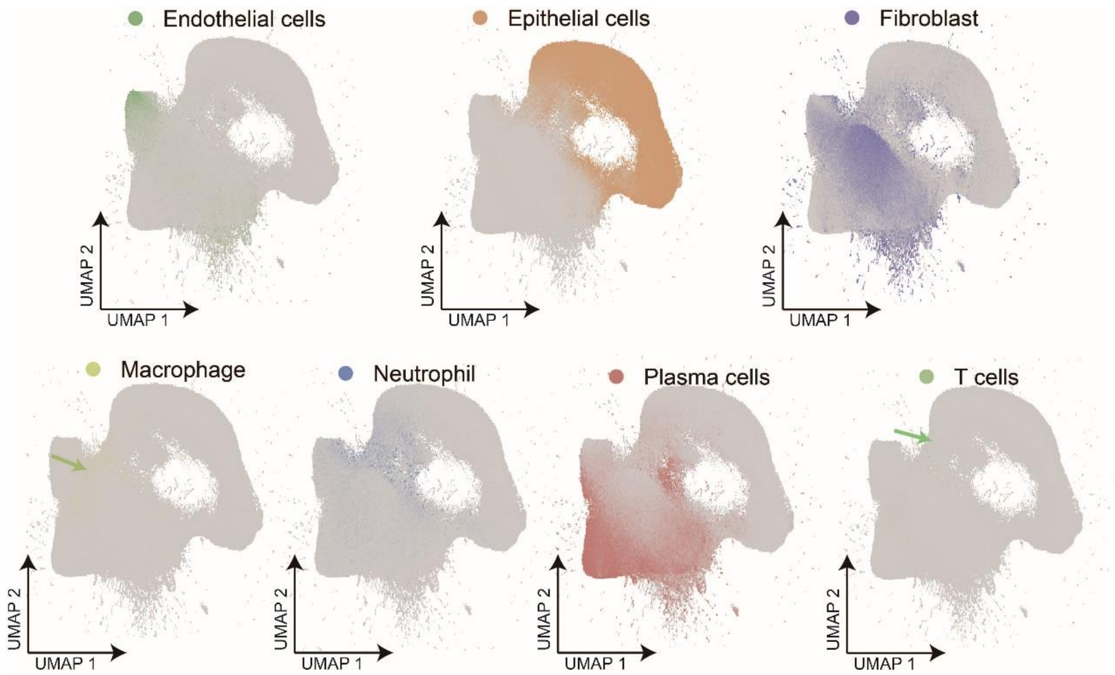


Sup Fig.1 Dot plots showing the expression of marker genes for the 7 major clusters (Epithelial cell, Macrophage, Endothelial cell, Plasma cell, Fibroblast, Neutrophil and T cell).

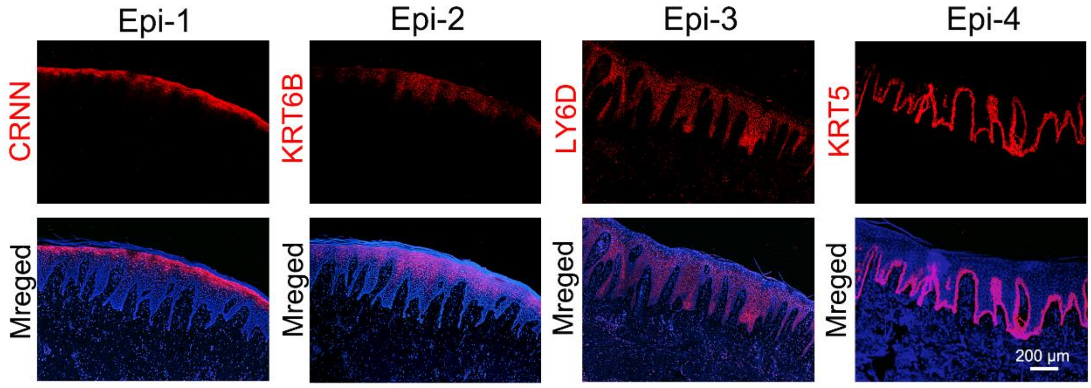


Sup Fig.2 Immunofluorescence staining shows different epithelial layers with single-cell genes markers (CRNN, KRT6B, LY6D, KRT5). Scale bar=200 μm.

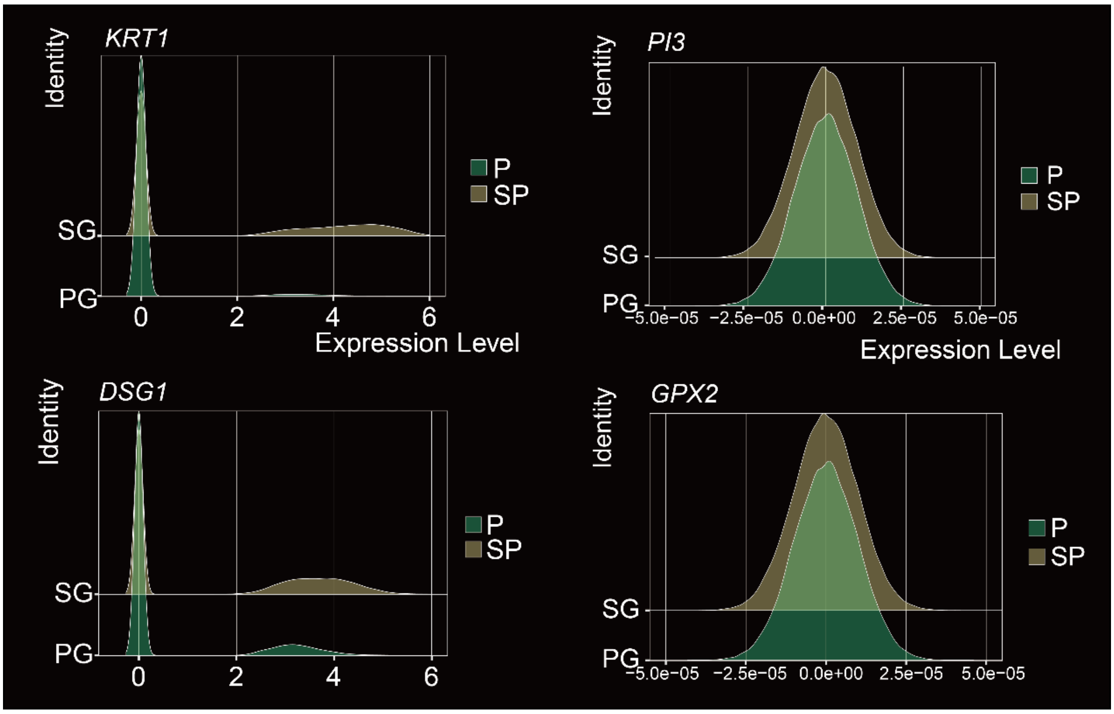


Sup Fig.3 Visium HD data analysis revealing the relative expression levels of *KRT1*, *DSG1*, *PI3*, and *GPX2* in the gingival tissue.

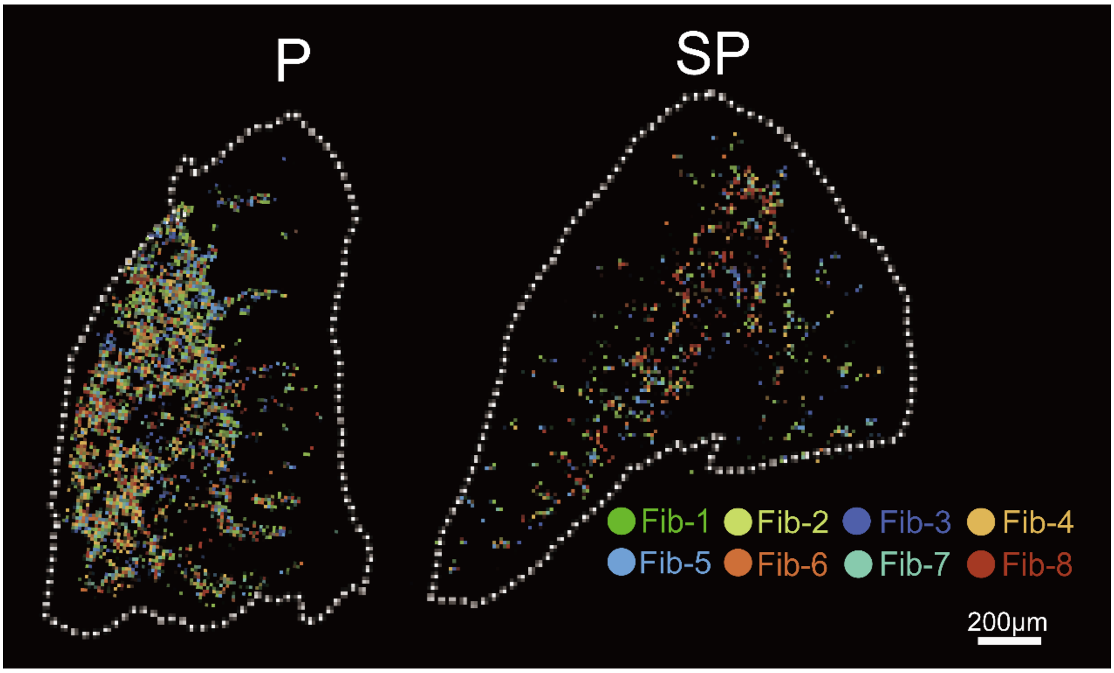


Sup Fig.4 Spatial map of the eight subgroups of fibroblasts in the Visium HD date. Scale bar=200 μm.

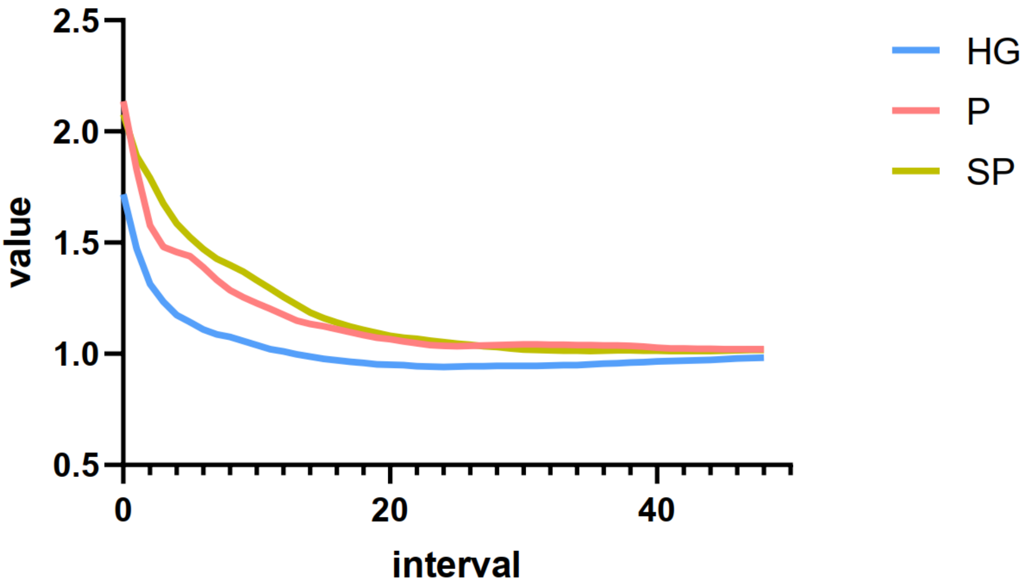


Sup Fig.5 The cell spatial relationship of macrophages and endothelial cells in the three group (HG、P、SP).

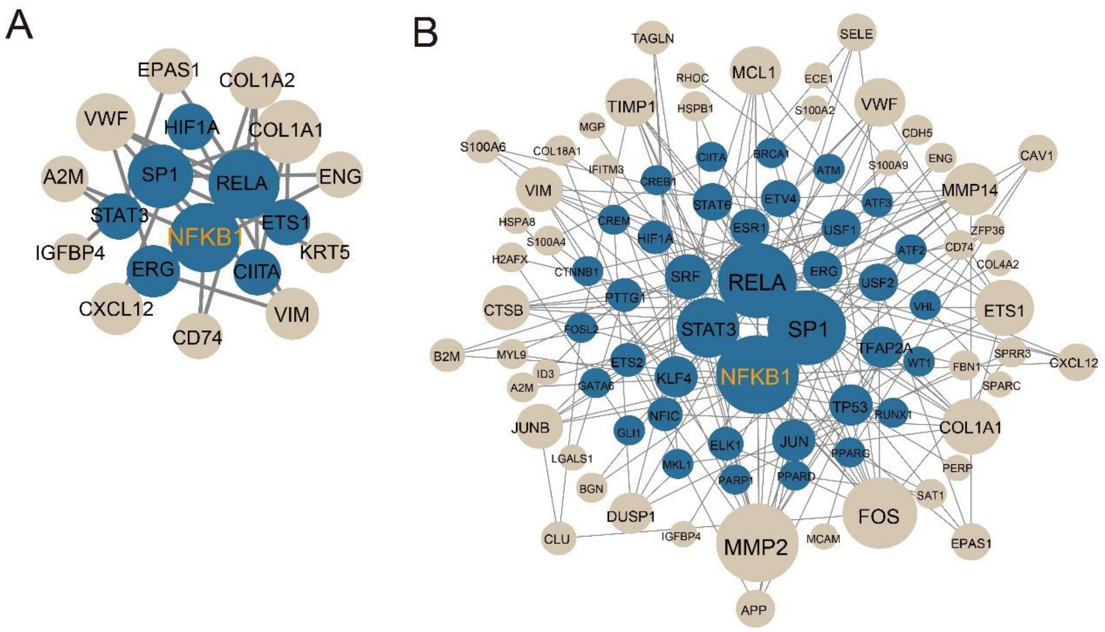


Sup Fig.6 (A). Gene regulatory network showing key transcription factors and upregulated genes in endothelial cells from the SP group compared with the HG group. (B). Gene regulatory network showing key transcription factors and upregulated genes in endothelial cells from the SP group compared with the P group.
